# Association Between Maternal Egg Consumption During Pregnancy and Breastfeeding Initiation and Duration †

**DOI:** 10.3390/nu17233710

**Published:** 2025-11-26

**Authors:** Xiaozhong Wen, Fatima Mohammed, Eve M. Giancarlo, Andrea Botchway, Daphkar Albert-Ducasse, Isabella Ritchie, Todd C. Rideout

**Affiliations:** 1 Division of Behavioral Medicine, Department of Pediatrics, Jacobs School of Medicine and Biomedical Sciences, State University of New York at Buffalo, Buffalo, NY 14214, USA; fmohamme@buffalo.edu (F.M.); evemgee@gmail.com (E.M.G.); abotchwa@buffalo.edu (A.B.); daphkara@buffalo.edu (D.A.-D.); isritchi@buffalo.edu (I.R.); 2 Department of Exercise and Nutrition Sciences, School of Public Health and Health Professions, State University of New York at Buffalo, Buffalo, NY 14214, USA; rideout@buffalo.edu

**Keywords:** egg, diet, nutrition, pregnancy, breastfeeding duration, breastfeeding initiation

## Abstract

**Background/Objectives**: Breastfeeding has positive effects on both maternal and offspring health. This study examined the association between egg consumption (total eggs and specific egg foods) and breastfeeding duration and initiation. **Methods**: Data from a U.S. cohort of 1039 mother–infant dyads in the Infant Feeding Practices Study II and its 6-year follow-up (2005–2012) were analyzed. In late pregnancy, mothers reported the frequency and serving size of their consumption of egg-related products in the past month, including total eggs, whole eggs, egg whites, egg substitutes, egg with fat, and egg salad. Multivariable logistic and linear regression models were used to examine associations of egg consumption with breastfeeding initiation and duration, respectively, adjusting for socio-demographics, pregnancy-related characteristics, and the Healthy Eating Index. **Results**: Mothers who consumed eggs 3+ times/week had higher odds of breastfeeding initiation (93.8% vs. 81.1%; confounder-adjusted OR, 3.34 [95% CI, 1.51–7.39]), compared to non-consumers. Similar associations were seen with whole eggs 2+ times/week (91.5% vs. 83.4%; 2.21 [95% CI, 1.20–4.04]), and eggs with fat 2+ times per week (91.4% vs. 86.8%; 2.19 [95% CI, 1.16–4.13]). Compared to non-consumers, mothers who consumed total eggs or whole eggs 1+ times per month had about 5 weeks longer breastfeeding duration, and those who consumed total eggs or whole eggs 1+ times per week had about 3 weeks longer exclusive breastfeeding duration. No significant associations were found for egg whites, egg salad, or egg substitutes. **Conclusions**: High consumption of total eggs, whole eggs, or eggs with fat may help support favorable breastfeeding practice outcomes.

## 1. Introduction

Breastfeeding has been shown to have positive effects on both maternal and offspring health. For mothers, breastfeeding is associated with a low risk of premenopausal breast cancer [1], ovarian cancer [2], postpartum weight retention [3], type 2 diabetes [4], and metabolic syndrome [5]. Psychologically, breastfeeding mothers report lower levels of anxiety, negative mood, stress, and depression compared to mothers who feed their infants with formula only [6]. For children, being breastfed is associated with better lung functioning and cognitive development, as well as a reduced risk for infections (e.g., otitis media, gastroenteritis, and pneumonia), obesity, type 2 diabetes, leukemia, sudden infant death syndrome (SIDS), asthma, and allergies [7,8]. Therefore, the American Academy of Pediatrics (AAP) [9] and American College of Obstetricians and Gynecologists (ACOG) [10] strongly advocate breastfeeding practices and recommend exclusive breastfeeding for the first 6 months of life, with continued breastfeeding as complementary foods are introduced through the infant’s first year of life, or longer as mutually desired by the mother and her infant. Although breastfeeding initiation is relatively high (e.g., 83.2% in 2019) in the U.S., continued breastfeeding is low (e.g., 55.8% at 6 months and 35.9% at 12 months), and the prevalence of exclusive breastfeeding is even lower (e.g., 24.9% at 6 months) [11]. Therefore, more interventions are needed to increase breastfeeding duration and promote exclusive breastfeeding.

Breastfeeding is a multifactorial behavior impacted by the biological ability of milk production, psychological factors, and environmental support systems [12]. Nutrition plays a crucial role in breastfeeding initiation and continuation, as a well-balanced diet can substantially improve both the volume and quality of breast milk [13]. For instance, as a nutrient-dense whole food offering complete protein along with essential vitamins and minerals (i.e., choline, vitamin D, vitamin B_12_, riboflavin, and selenium) [14,15], eggs consumed by mothers can potentially impact milk production. For example, in a study conducted in northern Thailand, a higher frequency of maternal consumption of egg and egg-related products (e.g., egg tofu and egg ball) was significantly correlated with (r = 0.361, *p*-value = 0.031) increased breast milk volume between 1 and 3 months postpartum among mothers who breastfed exclusively. In addition, both the frequency (r = 0.485, *p*-value < 0.01) and amount (r = 0.388, *p*-value < 0.05) of consuming egg tofu were positively correlated with breast milk volume, adjusting for infant birth weight, weight-for-age z-score, and maternal energy and carbohydrate intake [16]. Further, in another study, more than half (51.1%) of Chinese postpartum mothers reported consuming rice wine with egg soup, one of the popular galactagogue foods according to the traditional Chinese Medicine (e.g., replenishment of “Qi” and “Blood”) [17].

However, there is a lack of research on egg consumption and breastfeeding practices in the U.S. and other developed countries, where food supply and environment are substantially different from those in the developing countries (e.g., Thailand and China), where existing research in this field has been conducted. For example, the effect of egg consumption on breastfeeding practices in the U.S.—where alternative protein sources like dairy products and meats are more commonly consumed—may differ from that in developing countries. Thus, further research is needed to understand broader cultural and regional variations in egg consumption that may influence breastfeeding. In addition, there is a research gap in understanding how different egg components impact breastfeeding practices. It is well known that different parts of the egg have distinct nutritional profiles, e.g., egg whites contain high protein and little to no fat, while egg yolks contain fat-soluble vitamins (e.g., D, E, and A), omega-3 fatty acids, phospholipids, and cholesterol [18]. Lastly, different preparation methods may alter the nutritional profile of eggs through temperature manipulation (e.g., heating and freezing), added ingredients (e.g., fat, salt, and spices), and extended storage time [19,20,21]. These changes may subsequently impact the contribution of egg consumption to breast milk production and secretion. Surprisingly, the potentially different impacts of varied egg components and preparation methods have not been addressed in previous research on breastfeeding practices.

Therefore, using data from a U.S. mother–infant dyad cohort, this study aimed to (1) quantify the consumption of various egg foods with different egg components and preparation methods among U.S. mothers, (2) identify significant determinants of egg consumption, and (3) examine the associations of overall egg intake and consumption of different egg foods with breastfeeding initiation and duration. The comprehensive measures of egg consumption in this cohort enabled us to make an innovative contribution to breastfeeding research, especially by addressing the potentially differential roles of various egg foods. We previously reported the preliminary results of this study as an abstract at the Nutrient 2024 conference [22]. 

## 2. Materials and Methods

### 2.1. Participants and Setting

A secondary data analysis was conducted within the Infant Feeding Practices Study II (IFPS II, 2005–2012), a U.S. longitudinal birth cohort study directed by the U.S. Food and Drug Administration (FDA) and the Centers for Disease Control and Prevention (CDC) [23]. This study enrolled 4902 pregnancies from the third trimester of pregnancy and followed mother–child dyads through the first year postpartum in 2005–2007 [23] with the addition of a Year 6 Follow-Up (Y6FU) in 2012 [24]. Subjects were invited for postpartum follow-ups if the mother was 18 years or older, had a single pregnancy, and the infant was born at least 35 weeks of gestation.

For our analysis, a subsample of 1039 women from the IFPS II study was included. Subjects were considered eligible if they had complete data regarding egg consumption during pregnancy and breastfeeding practices. The study procedures were approved by the FDA’s Research Involving Human Subjects Committee and the U.S. Office of Management and Budget. Mothers signed consent forms to participate in the IFPS II/Y6FU. The University at Buffalo Institute Review Board approved this secondary data analysis with de-identified data.

**Figure A1:**
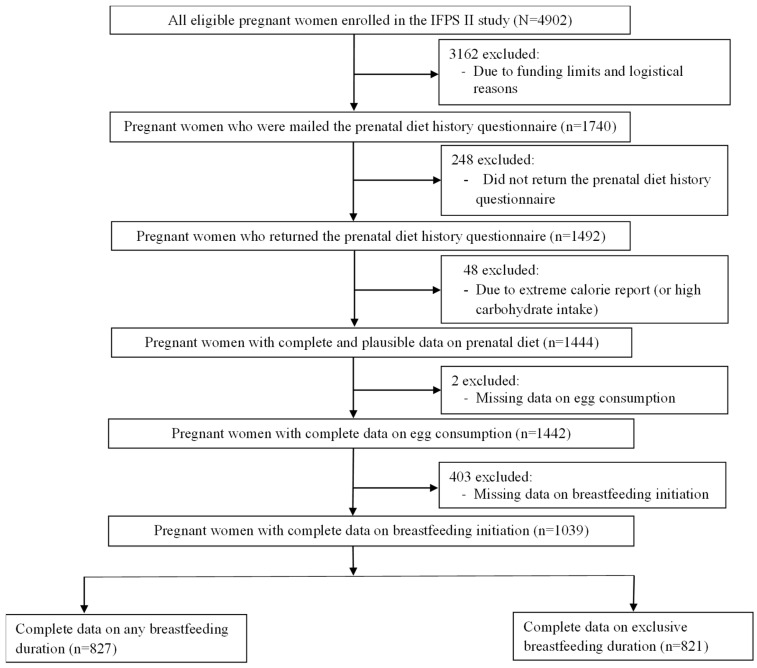
Sample Flow chart.

Appendix A Figure A1 shows the sample flow chart. Of the original 4902 pregnant women enrolled in IFPS II, 1442 had complete data on egg consumption during pregnancy (key exposure). Among them, 1039 had a known status of breastfeeding initiation (outcome #1), 827 had information on any breastfeeding duration (outcome #2), and 821 had information on exclusive breastfeeding duration (outcome #3). Overall, the distribution of sociodemographic and pregnancy characteristics was considerably different between the analytic sample (n = 1039) and the excluded sample (n = 3863) (Appendix A Table A1). Specifically, mothers in the analytic sample were older, had higher household-size-adjusted income (indicated by the percentage of federal poverty level) and higher education level, were more likely to be non-Hispanic White, have a household of 3 people, be non-recipients of the Special Supplemental Nutrition Program for Women, Infants, and Children (WIC), and be non-smokers, compared to mothers in the excluded sample. Pre-pregnancy body mass index (BMI), employment status, and residential regions were similar between the analytic and excluded subsamples.

### 2.2. Measures for Maternal Egg Consumption During Pregnancy

*Total egg consumption.* In IFPS II, pregnant mothers reported their frequency of consuming egg-related foods over the past month using a Diet History Questionnaire (DHQ). The egg-related foods included whole eggs, egg whites, egg substitutes, eggs with fat, and egg salad. The survey asked, “*Over the past month, how often did you eat eggs, egg whites, or egg substitutes (not counting eggs in baked goods and desserts)? (Please include eggs in salads, quiche, and souffles.)*”. The response options included: never, 1 time per month, 2–3 times per month, 1 time per week, 2 times per week, 3–4 times per week, 5–6 times per week, 1 time per day, and 2 or more times per day. Answers were recategorized into never, 1–3 times per month, 1–2 times per week, and 3 or more times per week to preserve the statistical power. For participants who reported consuming eggs, the next question assessed portion size: “*Each time you ate eggs, how many did you usually eat?*”. The response options for portion size included 1 egg (a standard portion), 2 eggs, and 3 or more eggs. The amount of eggs consumed was calculated using the mathematical formula: the frequency of egg consumption × portion size. The original frequency categories were converted to values in the same unit (times/week) to facilitate calculation, i.e., 0 times/week for never, 0.230137 times/week for 1 time per month, 0.575343 times/week for 2–3 times per month (mid-point), 1 time/week for 1 time per week, 2 times/week for 2 times per week, 3.5 times/week for 3–4 times per week (mid-point), 5.5 times/week for 5–6 times per week (mid-point), 7 times/week for 1 time per day, and 14 times/week for 2 or more times per day (the lower boundary for this high-frequency category). The original portion size category “3 or more eggs” was coded as “3 eggs” (the lower boundary for this high-portion category) to facilitate calculation.

*Whole eggs, egg whites, egg substitutes, egg with fat, and egg salad.* The proportion of consumed eggs as whole eggs was based on the following question: “*How often were the eggs you ate regular whole eggs?*”. The response options included almost never or never, about ¼ of the time, about ½ of the time, about ¾ of the time, and almost always or always. These proportion options were then converted to 0%, 25%, 50%, 75%, and 100%. To compute the amount of whole eggs consumed, the following formula was used: the amount of whole eggs consumed = the amount of total eggs consumed × the proportion of eggs consumed as whole eggs. They also reported the proportion of consumed eggs as egg whites, egg substitutes, egg with fat, or egg salad based on the following questions, respectively: “*How often were the eggs you ate egg whites only?*”, “*How often were the eggs you ate egg substitutes?*”, “*How often were the eggs you ate cooked in oil, butter, or margarine?*”, and “*How often were the eggs you ate part of egg salad?*” Their response options were the same as those for whole eggs, i.e., almost never or never, about ¼ of the time, about ½ of the time, about ¾ of the time, and almost always or always. Similarly, the amount of each egg food consumed was calculated as the amount of total eggs consumed × the proportion of eggs consumed as a specific egg food.

### 2.3. Breastfeeding Outcome Measures

*Breastfeeding initiation*. Our breastfeeding outcomes included breastfeeding initiation and breastfeeding duration (any or exclusive). The breastfeeding initiation variable was compiled from the original data repeatedly collected through multiple questionnaires, almost monthly, from 1 to 12 months postpartum. The survey question was, “*Did you ever breastfeed this baby (or feed this baby your pumped milk)?*” with response options of ‘yes’ and ‘no’. A response of ‘yes’ to this question was defined as breastfeeding initiation.

*Any breastfeeding duration*. Any breastfeeding duration was calculated for infants who received the mother’s breast milk. The questionnaire asked, “*Have you completely stopped breastfeeding and pumping milk for your baby?*” with response options of ‘yes’ and ‘no’. If yes, mothers were further asked, “*How old was your baby when you completely stopped breastfeeding and pumping milk?*” with a free-text response in the units of days, weeks, or months. Responses to these two questions across all available monthly surveys from 1 to 12 months were considered in the computation of any breastfeeding duration. For mothers who had missing responses to questions on breastfeeding termination, additional information on their current status of breastfeeding was obtained from the infant food frequency chart, “*In the past 7 days, how often was your baby fed each food listed below?*” with a free-text response in the units of feedings per day or feedings per week. The food options listed a variety of foods, such as breast milk, formula, cow milk, baby cereal, fruit, vegetables, and meat. Accordingly, the infant was considered to be breastfed if he/she was fed with breast milk in the past 7 days. For infants still breastfed at the time of the 12-month survey, additional information on their breastfeeding duration was obtained from the 6-year follow-up question: “*How old was this child when you completely stopped both breastfeeding and pumping milk for him or her?*” The response to this question was collected in units of weeks or months.

*Exclusively breastfeeding duration*. The exclusive breastfeeding duration variable was defined as the longest duration when the infant only received breast milk but no other food since birth. Similar to any breastfeeding duration, the infant’s breastfeeding status was checked repeatedly over time, as well as introduction to other foods or beverages, based on questionnaires completed almost monthly from 1 to 12 months. The computation of the exclusive breastfeeding duration was similar to that for any breastfeeding duration, except that another requirement of not consuming formula, solid food, or beverages was added.

### 2.4. Confounders

Based on our conceptual model and existing research [25,26], factors associated with both maternal egg consumption and breastfeeding were considered as potential confounders in this study. They included maternal age [27,28], race/ethnicity [28,29], the highest education level [30,31], employment status [32,33], household size [34,35], annual household income [36,37], WIC recipient status [38,39], region of residency [33,40], Healthy Eating Index (HEI), pre-pregnancy BMI, and smoking status during pregnancy [26,41]. Appendix A Table A2 provides detailed information on their variable types and measurement units or categories.

Using the U.S. Department of Agriculture (USDA) Dietary Guidelines for Americans, HEI-2005 consisted of 9 adequacy components: total fruit (5 points), whole fruit (5 points), total vegetables (5 points), dark green and orange vegetables and legumes (5 points), total grains (5 points), whole grains (5 points), milk (10 points), meat and beans (10 points), and oils (10 points) [42]. In addition, it consisted of 3 moderation components: saturated fat (10 points), sodium (10 points), and calories from solid fats, alcoholic beverages (e.g., beer, wine, and distilled spirits), and added sugars (20 points). The total score of HEI-2005 was 100, with a higher score representing a better-quality diet.

### 2.5. Statistical Analysis

In the descriptive analysis of the sample socio-demographic and pregnancy-related characteristics, categorical variables (e.g., race/ethnicity) were summarized by frequencies and percentages. The continuous variables (e.g., age) were summarized by mean ± standard deviation (SD). The distribution of these characteristics was compared using Chi-square tests for categorical variables or *t*-tests for continuous variables.

Analysis of variance (ANOVA) was conducted to quantify the overall differences in egg consumption by maternal characteristics, and Fisher’s least significant difference (LSD) test for pairwise comparisons between any two categories of these correlates. To identify potential correlates of breastfeeding initiation probability, the sample was analyzed using Chi-square (for categorical variables) and *t*-tests (for continuous variables). ANOVA and linear regression models were used to identify significant correlates of any or exclusive breastfeeding duration.

A multivariable logistic regression model was fitted to estimate the crude and adjusted odds ratios (ORs) and 95% confidence intervals (CIs) for the association between total egg consumption and breastfeeding initiation. The reference for the egg consumption variable was “never”. Linear regression models were used to estimate the crude and adjusted mean difference in breastfeeding duration in weeks by the frequency of total egg consumption or a one-egg/week increment of the amount of egg consumption. Similar analytic approaches were applied to examine the association of consuming each egg food (i.e., whole eggs, egg whites, egg substitutes, eggs with fat, and egg salad) with breastfeeding outcomes.

All data analyses were performed using SAS 9.4 (SAS Institute, Cary, NC, USA). Statistical significance was defined as a two-sided *p*-value < 0.05.

## 3. Results

### 3.1. Sample Characteristics

Table 1 shows the socio-demographic and pregnancy-related characteristics of the analytical sample (N = 1039). The average age was 29.02 years (SD, 5.34), the average% of the federal poverty level was 262.09% (SD, 189.28%), and the average pre-pregnancy BMI was 26.28 kg/m^2^ (SD, 6.45). The majority of pregnant mothers were Non-Hispanic White (85.2%), received a college education (81.1%), were employed (64.8%), had a household income level greater than $40,000 per year (56.9%), were not recipients of WIC (59.6%), and did not smoke during pregnancy (91.0%). The geographic regions ranged from 5.0% in New England to 21.7% in East North Central.

### 3.2. Correlates of Egg Consumption During Pregnancy

Table 2 displays the distribution of egg consumption frequency during pregnancy by maternal characteristics. The total egg consumption was 2.79 eggs/week among pregnant mothers. The most consumed egg foods were whole eggs (2.51 eggs/week), followed by eggs with fat (1.65 eggs/week), egg salad (0.23 eggs/week), egg white (0.19 eggs/week), and egg substitute (0.07 eggs/week). Egg consumption varied by race/ethnicity (*p*-value = 0.013), with the highest level among non-Hispanic Black mothers (3.95 eggs/week) and the lowest level among non-Hispanic White mothers (2.66 eggs/week). There was no significant difference in total egg consumption by other socio-demographics or smoking status.

Consumption of egg whites and egg substitutes did not vary significantly by any socio-demographics or smoking status. Non-Hispanic White mothers consumed fewer whole eggs (2.38 eggs/week) compared to Asian/Pacific Islander/Other mothers (3.53 eggs/week). Consumption of eggs with fat varied substantially by multiple correlates. Non-Hispanic Black mothers (3.30 eggs/week) consumed more eggs with fat than non-Hispanic White mothers (1.50 eggs/week) and Hispanic mothers (1.96 eggs/week). There was a decreasing trend in the consumption of eggs with fat with a higher maternal education level: for example, mothers with a high school or lower education consumed 2.07 eggs/week, while those who were college graduates consumed only 1.20 eggs/week. Similarly, maternal consumption of eggs with fat declined with annual household income, from 2.25 eggs/week in mothers with <$25,000/year to 1.33 eggs/week in mothers with ≥ $60,000/year. WIC recipients consumed more eggs with fat (2.03 eggs/week) than WIC non-recipients (1.39 eggs/week). Egg salad consumption only varied by race/ethnicity, with the highest level among non-Hispanic Black mothers (0.47 eggs/week) and the lowest level among non-Hispanic White mothers (0.20 eggs/week).

### 3.3. Distribution and Correlates of Breastfeeding Outcomes

In the total sample, 87.1% of mothers initiated breastfeeding. Table 3 demonstrates the probability of breastfeeding initiation by socio-demographic and pregnancy-related characteristics. Breastfeeding mothers had a higher mean% of the federal poverty level (mean difference, 34.35%; *p*-value = 0.0499) and lower pre-pregnancy BMI (mean difference, −1.64 kg/m^2^; *p*-value = 0.018) than non-breastfeeding mothers. The probability of breastfeeding initiation varied significantly (*p*-value < 0.001) across maternal education categories: 77.5% among mothers with high school or lower education, 86.3% among those with 1–3 years of college, 92.6% among those with college, and 91.4% among those with postgraduate education. Mothers who were WIC non-recipients (89.7%) had a higher probability of breastfeeding initiation compared to mothers who were recipients (83.3%) (*p*-value = 0.003). The probability of breastfeeding initiation varied significantly (*p*-value = 0.005) across the regions, for example, the highest (98.2%) in mothers from the Pacific region and the lowest (81.2%) in mothers from the Middle Atlantic region. Mothers who did not smoke during pregnancy (88.3%) had a higher probability of initiating breastfeeding compared to those who smoked during pregnancy (77.4%) (*p*-value = 0.003). There was no significant difference in breastfeeding initiation by maternal age, race/ethnicity, employment status, household size, or annual household income.

On average, mothers breastfed for 26.43 weeks, with exclusive breastfeeding duration being 6.31 weeks. Appendix A Table A3 shows the variation in any and exclusive breastfeeding duration by socio-demographic and pregnancy-related characteristics. Almost all characteristics were significant except for household size and child sex. For a one-year increment of maternal age, the mean difference for any breastfeeding was 1.09 weeks (*p*-value < 0.001) and 0.14 weeks for exclusive breastfeeding (*p*-value < 0.001). For a one-100% increment of the federal poverty level, the mean difference for any breastfeeding was 1.10 weeks (*p*-value < 0.001), while exclusive breastfeeding was 0.20 weeks (*p*-value = 0.017). Pre-pregnancy BMI was negatively associated with breastfeeding duration. For a one-unit increment of BMI, a mean difference was −0.28 weeks for any breastfeeding (*p*-value < 0.001) and −0.13 weeks for exclusive breastfeeding (*p*-value < 0.001). Non-Hispanic Asian/Pacific Islander/Other mothers had the longest any breastfeeding duration of 27.11 weeks, while Non-Hispanic Black mothers had the shortest any breastfeeding duration of 16.82 weeks. For exclusive breastfeeding, non-Hispanic Black mothers had the shortest duration, at 1.59 weeks, while non-Hispanic White mothers had the longest duration, at 5.79 weeks. Breastfeeding duration increased with maternal education (*p*-value < 0.001), for example, 15.76 weeks for mothers with high school or lower education, and 36.78 weeks for mothers with postgraduate education. Similarly, exclusive breastfeeding duration increased with maternal education (*p*-value < 0.001), for example, 3.32 weeks for mothers with high school or lower education, and 8.09 weeks for mothers with postgraduate education. Unemployed mothers had a longer breastfeeding duration than employed mothers, i.e., 27.17 vs. 23.30 weeks for any breastfeeding (*p*-value <0.001) and 6.24 vs. 4.87 weeks for exclusive breastfeeding (*p*-value < 0.001). Overall, both any and exclusive breastfeeding duration increased with household income (*p*-values < 0.001); for example, mothers with household income <$25,000 breastfed for 17.65 weeks and exclusively breastfed for 4.01 weeks, while mothers with household income ≥$60,000 breastfed for 27.49 weeks and exclusively breastfed for 5.59 weeks. WIC recipient status was another significant correlate, with non-recipients breastfeeding longer (27.23 vs. 21.03 weeks for any breastfeeding [*p*-value < 0.001] and 5.68 vs. 4.80 weeks for exclusive breastfeeding [*p*-value = 0.008]) than recipients. The breastfeeding durations varied significantly (*p* < 0.001) across the regions. For example, mothers in the Pacific region had the longest any breastfeeding duration (32.50 weeks) and exclusive breastfeeding duration (7.56 weeks). In comparison, mothers in the East South Central had the shortest any breastfeeding duration (18.67 weeks), and mothers in the Middle Atlantic region had the shortest exclusive breastfeeding duration (4.19 weeks). Non-smoking mothers breastfed longer (26.32 vs. 10.29 weeks for any breastfeeding and 5.71 vs. 1.92 weeks for exclusive breastfeeding, *p*-values <0.001) than cigarette-smoking mothers.

### 3.4. Association of Egg Consumption During Pregnancy with Breastfeeding Initiation

*Total egg consumption*. As shown in Table 4, pregnant individuals who consumed eggs 3 times per week or more had a significantly higher probability of breastfeeding initiation (93.8% vs. 81.1%; confounder-adjusted OR, 3.34 [95% CI, 1.51–7.39]; *p*-value = 0.003), compared to those who never consumed eggs. The probability of breastfeeding initiation increased with the amount of egg consumption, and the confounder-adjusted OR was 1.11 (95% CI, 1.03–1.20; *p*-value = 0.005) per 1 egg/week increment of egg consumption.

*Whole egg consumption*. Pregnant individuals who consumed whole eggs 2 times per week or more had a significantly higher probability of breastfeeding initiation (91.5% vs. 83.4%; confounder-adjusted OR, 2.21 [95% CI, 1.20–4.04]; *p*-value = 0.010) compared to those who never consumed whole eggs. The probability of breastfeeding initiation increased with the amount of whole egg consumption, and the confounder-adjusted OR was 1.13 (95% CI, 1.04–1.23; *p*-value = 0.004) per 1 egg/week increment of whole egg consumption.

*Egg white consumption*. The probability of breastfeeding initiation did not vary significantly by the frequency of egg white consumption: 87.3% among pregnant individuals who never consumed egg whites, 84.8% among those consuming egg whites 1–3 times per month, and 85.3% among those consuming egg whites 1 time per week or more. The probability of breastfeeding initiation did not change with the amount of egg white consumption, and the confounder-adjusted OR was 0.95 (95% CI, 0.79–1.13; *p*-value = 0.550) per 1 egg/week increment of egg white consumption.

*Egg substitute consumption*. Pregnant individuals who consumed egg substitutes had a similar probability of breastfeeding initiation (87.5% vs. 87.1) to those who never consumed egg substitutes. The probability of breastfeeding initiation did not significantly change with egg substitute consumption, and the confounder-adjusted OR was 1.09 (95% CI, 0.54–2.22; *p*-value = 0.804) per 1 egg/week increment of egg substitute consumption.

*Egg with fat consumption*. Pregnant individuals who consumed eggs with fat 2 times per week or more had a significantly higher probability of breastfeeding initiation (91.4% vs. 86.8%; confounder-adjusted OR, 2.19 [95% CI, 1.16–4.13]; *p*-value = 0.015), compared to those who never consumed eggs with fat. The probability of breastfeeding initiation increased with the amount of eggs with fat consumed, and the confounder-adjusted OR was 1.09 (95% CI, 1.00–1.19; *p*-value = 0.041) per 1 egg/week increment of consuming eggs with fat.

*Egg salad consumption*. Consuming egg salads 1–3 times per month (86.4% vs. 87.2) or 1 time per week or more (88.2% vs. 87.2%) was not significantly associated with the probability of breastfeeding initiation, compared to those who never consumed egg salads. Consistently, the probability of breastfeeding initiation did not increase with the amount of egg salad consumption, and the confounder-adjusted OR was 1.03 (95% CI, 0.77–1.38; *p*-value = 0.834) per 1 egg/week increment of egg salad consumption.

### 3.5. Association of Egg Consumption During Pregnancy with Any Breastfeeding Duration

*Total egg consumption.* Compared to pregnant individuals who never consumed eggs, those who consumed eggs 1–3 times per month (26.93 vs. 19.35 weeks; confounder-adjusted mean difference, 5.80 weeks [95% CI, 1.55–10.06]; *p*-value = 0.008), 1–2 times per week (27.76 vs. 19.35 weeks; 6.48 [95% CI, 2.18–10.78]; *p*-value = 0.003), 3 times per week or more (27.82 vs. 19.35 weeks; 5.16 [95% CI, 0.41–9.92]; *p*-value = 0.033) had a significantly higher mean breastfeeding duration (Table 5).

*Whole egg consumption.* Similarly, pregnant individuals who consumed whole eggs 1–3 times per month (26.84 vs. 21.35 weeks; adjusted mean difference, 4.80 weeks [95% CI, 0.98–8.61]; *p*-value = 0.014), 1 time per week (29.18 vs. 21.35 weeks; 6.41 weeks [95% CI, 1.81–11.00]; *p*-value = 0.006), or 2 times per week or more (27.33 vs. 21.35 weeks; 4.64 weeks [95% CI, 0.71–8.56]; *p*-value = 0.021) had a significantly higher mean breastfeeding duration, compared to those who never consumed whole eggs.

*Eggs with fat consumption.* Pregnant individuals who consumed eggs with fat 1–3 times per month had a significantly higher mean breastfeeding duration (27.77 vs. 26.00 weeks; adjusted mean difference, 3.51 weeks [95% CI, 0.37–6.66]; *p*-value = 0.029), compared to those who never consumed eggs with fat.

*Egg white, egg substitute, and egg salad consumption.* There were no significant associations of egg whites, egg substitutes, or egg salad with any breastfeeding duration.

### 3.6. Association of Egg Consumption During Pregnancy with Exclusive Breastfeeding Duration

*Total egg consumption.* Pregnant individuals who consumed eggs 1–2 times per week (7.25 vs. 3.64 weeks; adjusted mean difference, 2.91 weeks [95% CI, 0.89–4.93]; *p*-value = 0.005), or 3 times per week or more (7.69 vs. 3.64 weeks; adjusted mean difference, 2.84 weeks [95% CI, 0.61–5.07]; *p*-value = 0.013) had a significantly higher mean exclusive breastfeeding duration, compared to those who never consumed eggs (Table 6).

*Whole egg consumption.* Pregnant individuals who consumed whole eggs 1 time per week (7.88 vs. 4.26 weeks; adjusted mean difference, 3.21 weeks [95% CI, 1.05–5.38]; *p*-value = 0.004), or 2 times per week or more (7.13 vs. 4.26 weeks; 2.66 weeks [95% CI, 0.82–4.51]; *p*-value = 0.005) had a significantly higher mean exclusive breastfeeding duration, compared to those who never consumed whole eggs.

*Eggs with fat consumption.* Pregnant individuals who consumed eggs with fat 2 times per week or more had a significantly higher mean exclusive breastfeeding duration (7.21 vs. 5.95 weeks; adjusted mean difference, 2.06 weeks [95% CI, 0.31–3.81]; *p*-value = 0.021), compared to those who never consumed eggs with fat.

*Egg white, egg substitute, and egg salad consumption.* There were no significant associations of egg whites, egg substitutes, or egg salad with exclusive breastfeeding duration.

## 4. Discussion

In a national U.S. sample, this study investigated the relationship of overall egg consumption, as well as specific egg foods, distinguished by components or preparation methods, with breastfeeding initiation and duration. The results indicated that a higher consumption of total eggs, whole eggs, or eggs with fat was associated with a higher probability of breastfeeding initiation and longer breastfeeding duration (both any and exclusive). No such associations were found for egg whites, egg substitutes, or egg salad. These novel findings address current gaps in understanding the role of egg consumption in human lactation and can inform nutritional strategies to promote breastfeeding.

### 4.1. Distribution and Correlates of Maternal Egg Consumption

In this study sample, whole eggs were consumed the most, followed by eggs with fat, egg salad, egg whites, and egg substitutes. This consumption pattern is likely related to the simplicity and convenience of preparing whole eggs or eggs with fat (i.e., oil, butter, or margarine). Whole eggs offer a complete nutritional profile, containing fat-soluble vitamins, omega-3 fatty acids, phospholipids, and protein [18]. Thus, pregnant mothers may benefit from consuming whole eggs over other egg foods that contain an incomplete nutritional profile or have additional added ingredients (i.e., fat and sugar).

It was observed that egg consumption during pregnancy varied greatly by socio-demographic characteristics. In terms of race/ethnicity, non-Hispanic Black mothers consumed the most amount of total eggs, egg whites, eggs with fat, and egg salad. This high intake pattern aligns with previous research on egg consumption in non-pregnant individuals [43] and may be attributed to eggs being a cultural staple in the diet of the non-Hispanic Black population.

Our findings also suggested that mothers receiving WIC benefits consumed more eggs with fat compared to mothers not receiving WIC benefits. WIC food packages provide free access to eggs as an affordable non-meat source of protein, which can be used to make tasty and inexpensive meals (e.g., fried or scrambled eggs) along with added oil or butter. Educational materials provided by WIC, such as websites and brochures, also contain egg recipes, tips for using eggs in different dishes, and cooking instructions, further encouraging egg consumption.

In our sample, there was an overall decrease in the consumption of eggs with fat among mothers with higher education or annual household income. This may be because more affluent populations have greater accessibility to other costly protein options, such as meat and seafood (estimated to be twice as expensive as eggs per 100 g of protein [44]. Additionally, mothers with a high socio-economic status may be more health-conscious and aware of the potential harms of excessive fat consumption and therefore choose other egg preparation methods (i.e., hard boiling or poaching).

### 4.2. Associations Between Maternal Egg Consumption and Breastfeeding Outcomes

Overall, this study found that total egg consumption was associated with favorable breastfeeding practice outcomes, including a higher likelihood of breastfeeding initiation and a longer breastfeeding duration (both any and exclusive). Our findings were consistent with a previous study from northern Thailand, which reported an association of egg and egg tofu consumption with increased milk volume [16]. In traditional Chinese medicine, eggs are considered galactagogue foods that can stimulate milk production [17]. From a nutritional perspective, eggs are nutrient-dense, providing a substantial portion of the Recommended Dietary Allowance (RDA) for protein, lipids, and micronutrients. For example, two 50-g eggs can provide approximately 18% of the RDA of protein and 53% of the RDA of choline for lactating women [15]. By helping meet the increased nutritional needs of lactating mothers, egg consumption can biologically support breast milk production and secretion, and thus facilitate lactation initiation and continued breastfeeding practices for an extended duration. In addition, egg consumption may contribute to maternal health and well-being. For example, a previous study among Chinese mothers found that a 1 g increase in egg intake was associated with a 0.3 μg/mL increase in the concentration of breast milk lactoferrin, an iron-binding protein that protects against bacterial infections [45].

A particularly novel finding of our study was the observation that distinct types of egg foods were differentially associated with breastfeeding outcomes. Specifically, high consumption of whole eggs or eggs with fat, but not egg whites or egg substitutes, was associated with a higher probability of breastfeeding initiation and longer breastfeeding duration. Although the underlying reasons for these differential associations are not entirely clear, one possible explanation is that whole eggs and eggs with fat contain egg yolks, while egg substitutes and egg whites do not. Egg yolks are high in fat (e.g., 28.8 g out of 100 g [46]), including omega-3 fatty acids, cholesterol, and phospholipids, whereas egg whites only have a trace amount of fat content (<0.1 g out of 100 g [47]) [18]. In a short-term feeding study among exclusively breastfeeding Australian mothers between 6 and 24 weeks postpartum, consumption of a high-fat diet (adding 28 g fat to the control diet; 45% of energy from fat) over 3 days has been shown to modify the nutrient profile of breast milk by increasing its concentrations of lactose (*p*-value = 0.006) and triglycerides (mean difference of 3.05 g/dL, *p*-value < 0.001), compared to the period of consuming the control diet (38% of energy from fat) [48]. Moreover, a cross-sectional study in China found that maternal dietary fatty acid intake was positively correlated (*p* <0.001) with levels of saturated fatty acids, polyunsaturated fatty acids, and docosahexaenoic acids in breast milk [49]. A higher fat content of breast milk was correlated with a longer breastfeeding duration (R^2^ = 0.22, *p*-value < 0.001) in a previous study from Israel [50], partially due to some infants’ preference for high-fat milk [51]. Maternal intake of long-chain polyunsaturated fatty acids (LCPUFA), which are rich in eggs, also plays an essential role in mammary gland function [52]. For example, animal research suggests that an LCPUFA-deficient diet can impair the responsiveness of mammary epithelial cells to prolactin stimulation [53]. In addition, egg yolks contain high amounts of natural choline, which may also play an important role in breastfeeding practices as it helps enhance fetal/infant brain development and reduce the risk of birth defects [54]. Neurocognitively healthy infants are more likely to cooperate with the mother to initiate breastfeeding successfully through skin-to-skin contact, chest navigation, locating the breasts, identifying the nipple, appropriate latching, effective nipple suckling, and breast stimulation with hand movements (e.g., grabbing, pulling, and pinching) [55]. Continued breastfeeding has higher requirements of infant motor skills, suckling force, muscle strength, and alertness than formula feeding from bottles [56].

### 4.3. Limitations and Strengths

Our study had several limitations. First, a reliance on self-reported diet history in the past month could lead to recall bias due to memory issues and social desirability concerns. Second, the sample consisted mainly of non-Hispanic White mothers with relatively high socioeconomic status, which could limit the external validity of our findings. Third, despite the IFPS II sample being nationally distributed, it was not nationally representative due to non-random sampling in the U.S. population. Therefore, our results might not be generalizable to the total population and should be interpreted with caution. Fourthly, this observational research could not prove causality. Egg consumption may likely serve as a proxy for a healthier diet or lifestyle rather than a cause of better breastfeeding outcomes.

However, several strengths of our study warrant mention. First, to our best knowledge, this study was the first to assess the associations between specific egg foods (with different egg components and preparation methods) and breastfeeding practices, providing insights into the roles of egg consumption in breastfeeding. Second, the IFPS II collected detailed information on infant feeding practices, which allowed us to comprehensively assess breastfeeding outcomes, including initiation, any breastfeeding duration, and exclusive breastfeeding duration. Third, adjusting for socio-demographics, pregnancy-related characteristics, and overall dietary quality improved the validity of our analytic results by reducing potential confounding bias.

## 5. Conclusions

In conclusion, our findings suggested that high consumption of total eggs, whole eggs, or eggs with fat was associated with more favorable breastfeeding practices (i.e., higher initiation and longer duration) among U.S. mothers. In contrast, consumption of egg whites or egg substitutes was not a significant determinant of breastfeeding. These novel findings fill some research gaps in this field by separating different egg foods based on egg components and preparation methods. Nevertheless, caution is needed when interpreting our findings due to recall bias, selection bias, residual confounding, and the observational study design (non-causal). Future research should utilize more valid dietary measurements, a larger and more diverse sample, and a randomized controlled intervention. Along with existing evidence, these findings can contribute to a better understanding of how egg consumption may contribute to breastfeeding and inform nutritional strategies to promote maternal and child health.

## Figures and Tables

**Table 1:** Socio-demographic and pregnancy-related characteristics of participants in the analytic sample.

	Analytic Sample (N = 1039)	
Characteristics *	n (%)	Mean ± SD
**Maternal age, years**		29.02 ± 5.34
**% of Federal poverty level**		262.09 ± 189.28
**Pre-pregnancy BMI, kg/m^2^**		26.28 ± 6.45
**Maternal race/ethnicity**		
Non-Hispanic White	880 (85.2)	
Non-Hispanic Black	40 (3.9)	
Hispanic	66 (6.4)	
Non-Hispanic Asian/Pacific Islander/Other	47 (4.6)	
**Maternal highest education level**		
High school or lower	187 (18.9)	
1–3 years of college	388 (39.2)	
College graduate	309 (31.2)	
Postgraduate	105 (10.6)	
**Maternal employment status**		
Unemployed	364 (35.2)	
Employed	671 (64.8)	
**Household size**		
1–2 people	260 (25.0)	
3 people	400 (38.5)	
4 people	217 (20.9)	
≥5 people	162 (15.6)	
**Annual household income level**		
<$25,000	220 (21.2)	
$25,000 ≤ $39,999	228 (21.9)	
$40,000 ≤ $59,999	243 (23.4)	
≥$60,000	348 (33.5)	
**Maternal WIC recipient status**		
Non-recipient	619 (59.6)	
Recipient	420 (40.4)	
**Region of residency**		
New England	52 (5.0)	
Middle Atlantic	122 (11.7)	
East North Central	225 (21.7)	
West North Central	96 (9.2)	
South Atlantic	153 (14.7)	
East South Central	56 (5.4)	
West South Central	117 (11.3)	
Mountain	107 (10.3)	
Pacific	111 (10.7)	
**Maternal cigarette smoking during pregnancy**		
No	940 (91.0)	
Yes	93 (9.0)	

BMI, body mass index; WIC, Special Supplemental Nutrition Program for Women, Infants, and Children; SD, standard deviation. * For some characteristics, the sum of categories is less than the total due to missing data.

**Table 2:** Maternal egg consumption during pregnancy by socio-demographic and pregnancy-related characteristics.

	Total Egg				Whole Egg				Egg White			
Characteristics	n	Mean ± SD (Eggs/Week)	Overall *p*-Value *	Pairwise Comparisons **	n	Mean ± SD (Eggs/Week)	Overall *p*-Value *	Pairwise Comparisons **	n	Mean ± SD (Eggs/Week)	Overall *p*-Value *	Pairwise Comparisons **
**Overall**	1038	2.79 ± 3.39			1037	2.51 ± 3.22			1037	0.19 ± 1.05		
**Maternal race/ethnicity**			**0.013**				**0.019**				0.310	
Non-Hispanic White	879	2.66 ± 3.25		a	878	2.38 ± 3.10		a	878	0.16 ± 0.96		
Non-Hispanic Black	40	3.95 ± 5.22		b	40	3.29 ± 4.72		a,b	40	0.46 ± 1.71		
Hispanic	66	3.10 ± 3.40		a,b	66	3.01 ± 3.38		a,b	66	0.24 ± 1.33		
Non-Hispanic Asian/Pacific Islander/Other	47	3.79 ± 3.53		b	47	3.53 ± 3.51		b	47	0.24 ± 0.75		
**Maternal education level**			0.500				0.330				0.603	
High school or lower	187	2.86 ± 3.99			187	2.59 ± 3.98			187	0.11 ± 0.56		
1–3 years of college	387	2.94 ± 3.46			387	2.71 ± 3.36			387	0.19 ± 1.05		
College graduate	309	2.55 ± 2.98			308	2.29 ± 2.69			309	0.19 ± 1.16		
Postgraduate	105	2.77 ± 3.11			105	2.31 ± 2.79			104	0.27 ± 1.26		
**Maternal employment status**			0.180				0.260				0.632	
Unemployed	364	2.99 ± 3.62			364	2.67 ± 3.50			364	0.21 ± 1.16		
Employed	670	2.69 ± 3.25			669	2.43 ± 3.07			669	0.18 ± 0.99		
**Household size**			0.840				0.664				0.999	
1–2 people	259	2.81 ± 3.34			259	2.52 ± 3.18			259	0.19 ± 1.05		
3 people	400	2.69 ± 3.33			400	2.38 ± 3.10			399	0.19 ± 1.12		
4 people	217	2.95 ± 3.42			216	2.72 ± 3.37			217	0.20 ± 0.96		
≥5 people	162	2.81 ± 3.59			162	2.56 ± 3.43			162	0.19 ± 0.97		
**Annual household income level**			0.514				0.279				0.426	
<$25,000	220	3.00 ± 3.99			220	2.78 ± 3.89			220	0.27 ± 1.37		
$25,000 ≤ $39,999	228	2.87 ± 3.09			227	2.64 ± 2.97			228	0.13 ± 0.73		
$40,000 ≤ $59,999	242	2.83 ± 3.61			242	2.50 ± 3.43			242	0.22 ± 1.21		
≥$60,000	348	2.59 ± 2.97			348	2.27 ± 2.73			347	0.16 ± 0.84		
**Maternal WIC recipient status**			0.116				0.063				0.446	
Non-recipient	618	2.66 ± 3.08			618	2.36 ± 2.88			617	0.17 ± 0.98		
Recipient	420	2.99 ± 3.79			419	2.74 ± 3.67			420	0.22 ± 1.14		
**Region of residency**			0.390				0.469				0.237	
New England	52	3.01 ± 3.93		a,b	52	2.54 ± 3.25		a,b	52	0.44 ± 2.34		a
Middle Atlantic	122	3.00 ± 3.36		a.b	122	2.56 ± 3.06		a,b	122	0.33 ± 1.37		a,c
East North Central	225	2.61 ± 2.94		a	225	2.33 ± 2.85		a,b	225	0.16 ± 0.81		a,b
West North Central	96	2.22 ± 2.93		a	96	2.06 ± 2.83		a	96	0.03 ± 0.13		b
South Atlantic	153	2.69 ± 3.32		a,b	152	2.34 ± 3.08		a,b	153	0.27 ± 1.27		a,b
East South Central	56	2.54 ± 2.98		a,b	56	2.26 ± 2.95		a,b	56	0.16 ± 0.97		a,b
West South Central	116	2.94 ± 3.67		a,b	116	2.80 ± 3.65		a,b	116	0.10 ± 0.57		b,c
Mountain	107	2.86 ± 3.99		a,b	107	2.81 ± 4.00		a,b	107	0.09 ± 0.70		b,c
Pacific	111	3.41 ± 3.68		b	111	2.99 ± 3.43		b	110	0.23 ± 0.87		a,b
**Maternal cigarette smoking during pregnancy**			0.164				0.275				0.127	
No	939	2.85 ± 3.42			938	2.55 ± 3.25			938	0.21 ± 1.10		
Yes	93	2.33 ± 3.07			93	2.17 ± 3.08			93	0.03 ± 0.31		
	**Egg Substitute**				**Egg with Fat**				**Egg Salad**			
**Characteristics**	**n**	**Mean ± SD** **(Eggs/Week)**	**Overall** *p*-Value *	**Pairwise** Comparisons **	**n**	**Mean ± SD** **(Eggs/Week)**	**Overall** *p*-Value *	**Pairwise** Comparisons **	**n**	**Mean ± SD** **(Eggs/Week)**	**Overall** *p*-Value *	**Pairwise** Comparisons **
**Overall**	1038	0.07 ± 0.45			1038	1.65 ± 2.84			1036	0.23 ± 0.74		
**Maternal race/ethnicity**			0.737				**<0.001**				**0.043**	
Non-Hispanic White	879	0.08 ± 0.48			879	1.50 ± 2.65		a	877	0.20 ± 0.62		a
Non-Hispanic Black	40	0.03 ± 0.16			40	3.30 ± 4.99		b	40	0.47 ± 1.51		b
Hispanic	66	0.04 ± 0.21			66	1.96 ± 2.82		a,c	66	0.22 ± 0.69		a,b
Non-Hispanic Asian/Pacific Islander/Other	47	0.04 ± 0.26			47	2.48 ± 3.33		b,c	47	0.41 ± 1.43		a,b
**Maternal education level**			0.275				**0.002**				0.927	
High school or lower	187	0.03 ± 0.16			187	2.07 ± 3.45		a	187	0.25 ± 0.82		
1–3 years of college	387	0.08 ± 0.55			387	1.85 ± 3.23		a	386	0.21 ± 0.76		
College graduate	309	0.06 ± 0.40			309	1.20 ± 1.94		b	308	0.23 ± 0.74		
Postgraduate	105	0.13 ± 0.50			105	1.44 ± 2.19		a,b	105	0.20 ± 0.59		
**Maternal employment status**			0.314				0.322				0.424	
Unemployed	364	0.09 ± 0.59			364	1.77 ± 2.89			364	0.20 ± 0.66		
Employed	670	0.06 ± 0.35			670	1.59 ± 2.81			668	0.24 ± 0.77		
**Household size**			0.091				0.309				0.764	
1-2 people	259	0.12 ± 0.64		a	259	1.74 ± 2.84			259	0.23 ± 0.63		
3 people	400	0.08 ± 0.46		a,b	400	1.45 ± 2.71			399	0.20 ± 0.77		
4 people	217	0.03 ± 0.18		b	217	1.87 ± 3.27			217	0.24 ± 0.73		
≥5 people	162	0.04 ± 0.27		a,b	162	1.72 ± 2.50			161	0.26 ± 0.82		
**Annual household income level**			0.306				**0.002**				0.770	
<$25,000	220	0.06 ± 0.50			220	2.25 ± 3.91		a	220	0.21 ± 0.88		
$25,000 ≤ $39,999	228	0.03 ± 0.29			228	1.58 ± 2.45		b	227	0.20 ± 0.50		
$40,000 ≤ $59,999	242	0.08 ± 0.53			242	1.64 ± 2.76		b	241	0.26 ± 0.88		
≥$60,000	348	0.10 ± 0.44			348	1.33 ± 2.21		b	348	0.23 ± 0.65		
**Maternal WIC recipient status**			0.080				**<0.001**				0.703	
Non-recipient	618	0.09 ± 0.48			618	1.39 ± 2.36		a	617	0.23 ± 0.73		
Recipient	420	0.04 ± 0.39			420	2.03 ± 3.39		b	419	0.21 ± 0.74		
**Region of residency**			0.350				0.705				0.379	
New England	52	0.04 ± 0.24		a,b	52	1.18 ± 2.79			51	0.39 ± 0.70		a
Middle Atlantic	122	0.07 ± 0.33		a,b	122	1.79 ± 2.80			122	0.24 ± 0.56		a,b
East North Central	225	0.06 ± 0.29		a,b	225	1.61 ± 2.55			225	0.21 ± 0.76		a,b
West North Central	96	0.05 ± 0.37		a,b	96	1.44 ± 2.55			96	0.10 ± 0.38		b
South Atlantic	153	0.13 ± 0.66		a,b	153	1.56 ± 2.89			153	0.23 ± 0.73		a,b
East South Central	56	0.03 ± 0.16		a,b	56	1.61 ± 2.79			56	0.19 ± 0.48		a,b
West South Central	116	0.06 ± 0.50		a,b	116	1.76 ± 3.02			115	0.25 ± 0.77		a,b
Mountain	107	0.03 ± 0.20		b	107	1.59 ± 2.86			107	0.15 ± 0.52		a,b
Pacific	111	0.16 ± 0.73		a	111	2.10 ± 3.42			111	0.32 ± 1.20		a
**Maternal cigarette smoking during pregnancy**			0.095				0.850				0.328	
No	939	0.08 ± 0.47			939	1.65 ± 2.86			937	0.23 ± 0.76		
Yes	93	0.00 ± 0.00			93	1.71 ± 2.75			93	0.15 ± 0.36		

SD, standard deviation. * Analysis of variance (ANOVA). Significant *p*-values (<0.05) are in bold. ** Least significant difference (LSD) test. If two groups do not share any letters, they have significantly different mean values of egg consumption.

**Table 3:** Probability of breastfeeding initiation by socio-demographic and pregnancy-related characteristics.

	Probability of Breastfeeding Initiation		
Characteristics *	n (%)	Mean Difference ± SE **	*p*-Value ***
**Overall**	905 (87.1)		
**Maternal age, years**		0.27 ± 0.49	0.581
**% of Federal poverty level**		34.35 ± 17.50	**0.050**
**Pre-pregnancy BMI, kg/m^2^**		−1.64 ± 0.60	**0.018**
**Maternal race/ethnicity**			0.088
Non-Hispanic White	762 (86.6)		
Non-Hispanic Black	32 (80.0)		
Hispanic	62 (93.9)		
Non-Hispanic Asian/Pacific Islander/Other	44 (93.6)		
**Maternal highest education level**			**<0.001**
High school or lower	145 (77.5)		
1–3 years of college	335 (86.3)		
College graduate	286 (92.6)		
Postgraduate	96 (91.4)		
**Maternal employment status**			0.183
Unemployed	310 (85.2)		
Employed	591 (88.1)		
**Household size**			0.122
1–2 people	236 (90.8)		
3 people	347 (86.8)		
4 people	181 (83.4)		
≥5 people	141 (87.0)		
**Annual household income level**			0.164
<$25,000	182 (82.7)		
$25,000 ≤ $39,999	200 (87.7)		
$40,000 ≤ $59,999	213 (87.7)		
≥$60,000	310 (89.1)		
**Maternal WIC recipient status**			**0.003**
Non-recipient	555 (89.7)		
Recipient	350 (83.3)		
**Region of residency**			**0.005**
New England	44 (84.6)		
Middle Atlantic	99 (81.2)		
East North Central	192 (85.3)		
West North Central	84 (87.5)		
South Atlantic	139 (90.9)		
East South Central	46 (82.1)		
West South Central	97 (82.9)		
Mountain	95 (88.8)		
Pacific	109 (98.2)		
**Maternal cigarette smoking during pregnancy**			**0.003**
No	830 (88.3)		
Yes	72 (77.4)		

BMI, body mass index; WIC, Special Supplemental Nutrition Program for Women, Infants, and Children; SE, standard error. * For some characteristics, the sum of categories is less than the total due to missing data. ** Mean difference = mean value of the characteristic of mothers with breastfeeding initiation—mean value of the characteristic of mothers without breastfeeding initiation. *** *p*-values from Chi-square tests for categorical correlates and *t*-test for continuous correlates. Significant *p*-values (<0.05) are in bold.

**Table 4:** Associations between maternal egg consumption during pregnancy and the probability of breastfeeding initiation.

Maternal Egg Consumption During Pregnancy	Probability of Breastfeeding Initiation				
	n (%)	Crude OR (95% CI)	Crude *p*-Value *	Adjusted OR (95% CI) **	Adjusted *p*-Value *
**Frequency of total egg consumption**					
Never	103 (81.1)	Reference		Reference	
1–3 times per month	319 (86.5)	1.49 (0.87–2.54)	0.146	1.47 (0.82–2.62)	0.195
1–2 times per week	302 (86.3)	1.47 (0.86–2.51)	0.164	1.34 (0.75–2.39)	0.327
3 times per week or more	181 (93.8)	**3.51 (1.69–7.32)**	**<0.001**	**3.34 (1.51–7.39)**	**0.003**
**Amount of egg consumption, 1 egg/week increment**		**1.11 (1.03–1.19)**	**0.006**	**1.11 (1.03–1.20)**	**0.005**
**Frequency of whole egg consumption**					
Never	141 (83.4)	Reference		Reference	
1–3 times per month	327 (85.6)	1.18 (0.72–1.94)	0.512	1.25 (0.73–2.13)	0.421
1 time per week	135 (85.4)	1.17 (0.64–2.12)	0.617	0.99 (0.52–1.87)	0.972
2 times per week or more	301 (91.5)	**2.13 (1.22–3.74)**	**0.008**	**2.21 (1.20–4.04)**	**0.010**
**Amount of whole egg consumption, 1 egg/week increment**		**1.12 (1.03–1.21)**	**0.006**	**1.13 (1.04–1.23)**	**0.004**
**Frequency of egg white consumption**					
Never	836 (87.3)	Reference		Reference	
1–3 times per month	39 (84.8)	0.81 (0.36–1.86)	0.624	0.42 (0.17–1.03)	0.058
1 time per week or more	29 (85.3)	0.85 (0.32–2.23)	0.736	0.49 (0.17–1.43)	0.193
**Amount of egg white consumption, 1 egg/week increment**		1.01 (0.84–1.21)	0.892	0.95 (0.79–1.13)	0.550
**Frequency of egg substitute consumption**					
Never	856 (87.1)	Reference		Reference	
Ever consumption	49 (87.5)	1.04 (0.46–2.34)	0.927	0.61 (0.25–1.46)	0.266
**Amount of egg substitute consumption, 1 egg/week increment**		1.50 (0.72–3.14)	0.283	1.09 (0.54–2.22)	0.804
**Frequency of eggs with fat consumption**					
Never	341 (86.8)	Reference		Reference	
1–3 times per month	293 (86.4)	0.97 (0.63–1.49)	0.894	1.12 (0.70–1.77)	0.643
1 time per week	81 (81.8)	0.69 (0.38–1.24)	0.210	0.74 (0.39–1.39)	0.347
2 times per week or more	190 (91.4)	1.61 (0.92–2.83)	0.098	**2.19 (1.16–4.13)**	**0.015**
**Amount of eggs with fat consumption, 1 egg/week increment**		1.04 (0.97–1.12)	0.259	**1.09 (1.00–1.19)**	**0.041**
**Frequency of egg salad consumption**					
Never	708 (87.2)	Reference		Reference	
1–3 times per month	165 (86.4)	0.93 (0.59–1.48)	0.766	1.04 (0.63–1.72)	0.878
1 time per week or more	30 (88.2)	1.10 (0.38–3.19)	0.858	1.07 (0.35–3.34)	0.901
**Amount of egg salad consumption, 1 egg/week increment**		1.02 (0.79–1.32)	0.865	1.03 (0.77–1.38)	0.834

OR, odds ratio; CI, confidence interval. * Significant *p*-values (<0.05) are in bold. ** Adjusted for household poverty level, maternal pre-pregnancy body mass index, Healthy Eating Index, race, education, employment status, enrollment in the Special Supplemental Nutrition Program for Women, Infants, and Children, region of residency, and cigarette smoking status during pregnancy.

**Table 5:** Associations between maternal egg consumption during pregnancy and any breastfeeding duration.

Maternal Egg Consumption During Pregnancy	Any Breastfeeding Duration, Weeks					
	Sample Size	Mean ± SD	Crude Mean Difference (95% CI)	Crude *p*-Value *	Adjusted Mean Difference (95% CI) **	Adjusted *p*-Value *
**Frequency of total egg consumption**						
Never	102	19.35 ± 19.91	Reference		Reference	
1–3 times per month	301	26.93 ± 20.60	**7.58 (2.98–12.18)**	**0.001**	**5.80 (1.55–10.06)**	**0.008**
1–2 times per week	270	27.76 ± 20.57	**8.41 (3.74–13.08)**	**<0.001**	**6.48 (2.18–10.78)**	**0.003**
3 times per week or more	154	27.82 ± 20.82	**8.47 (3.34–13.60)**	**0.001**	**5.16 (0.41–9.92)**	**0.033**
**Amount of total egg consumption, 1 egg/week increment**			0.18 (−0.23–0.58)	0.396	0.13 (−0.25–0.51)	0.497
**Frequency of whole egg consumption**						
Never	136	21.35 ± 20.56	Reference		Reference	
1–3 times per month	310	26.84 ± 20.67	**5.48 (1.34–9.62)**	**0.009**	**4.80 (0.98–8.61)**	**0.014**
1 time per week	121	29.18 ± 21.23	**7.82 (2.79–12.86)**	**0.002**	**6.41 (1.81–11.00)**	**0.006**
2 times per week or more	260	27.33 ± 20.20	**5.97 (1.71–10.23)**	**0.006**	**4.64 (0.71–8.56)**	**0.021**
**Amount of whole egg consumption, 1 egg/week increment**			0.15 (−0.28–0.57)	0.495	0.16 (−0.23–0.55)	0.413
**Frequency of egg white consumption**						
Never	761	26.37 ± 20.64	Reference		Reference	
1–3 times per month	39	29.32 ± 20.20	2.96 (−3.69–9.60)	0.383	−0.65 (−6.83–5.52)	0.836
1 time per week or more	26	22.97 ± 22.79	−3.39 (−11.46–4.68)	0.410	−5.93 (−13.46–1.61)	0.123
**Amount of egg white consumption, 1 egg/week increment**			−0.35 (−1.91–1.22)	0.666	−0.90 (−2.41–0.60)	0.240
**Frequency of egg substitute consumption**						
Never	780	26.34 ± 20.59	Reference		Reference	
Ever consumption	47	27.99 ± 22.41	1.66 (−4.43–7.74)	0.593	−2.74 (−8.50–3.02)	0.351
**Amount of egg substitute consumption, 1 egg/week increment**			2.72 (−0.21–5.65)	0.069	−0.05 (−2.85–2.74)	0.971
**Frequency of eggs with fat consumption**						
Never	314	26.00 ± 20.92	Reference		Reference	
1–3 times per month	272	27.77 ± 21.15	1.77 (−1.59–5.12)	0.302	**3.51 (0.37–6.66)**	**0.029**
1 time per week	78	25.59 ± 20.04	−0.42 (−5.54–4.71)	0.874	1.70 (−3.01–6.41)	0.479
2 times per week or more	163	25.42 ± 19.82	−0.58 (−4.49–3.33)	0.770	1.51 (−2.19–5.21)	0.423
**Amount of eggs with fat consumption, 1 egg/week increment**			−0.27 (−0.76–0.21)	0.274	0.02 (−0.44–0.48)	0.938
**Frequency of egg salad consumption**						
Never	660	26.28 ± 20.84	Reference		Reference	
1–3 times per month	139	26.19 ± 20.21	−0.10 (−3.88–3.68)	0.959	0.89 (−2.62–4.39)	0.620
1 time per week or more	26	31.28 ± 19.85	4.99 (−3.11–13.09)	0.227	5.98 (−1.55–13.50)	0.120
**Amount of egg salad consumption, 1 egg/week increment**			0.95 (−1.09–2.99)	0.363	1.18 (−0.70–3.07)	0.219

SD, standard deviation; CI, confidence interval. * Significant *p*-values (<0.05) are in bold. ** Adjusted for household poverty level, maternal pre-pregnancy body mass index, Healthy Eating Index, race, education, employment status, enrollment in the Special Supplemental Nutrition Program for Women, Infants, and Children, region of residency, and cigarette smoking status during pregnancy.

**Table 6:** Associations between maternal egg consumption during pregnancy and exclusive breastfeeding duration.

Maternal Egg Consumption During Pregnancy	Exclusive Breastfeeding Duration, Weeks					
	Sample Size	Mean ± SD	Crude Mean Difference (95% CI)	Crude *p*-Value *	Adjusted Mean Difference (95% CI) **	Adjusted *p*-Value *
**Frequency of total egg consumption**						
Never	102	3.64 ± 7.29	Reference		Reference	
1–3 times per month	301	5.69 ± 8.99	2.06 (−0.03–4.14)	0.053	1.51 (−0.48–3.51)	0.137
1–2 times per week	265	7.25 ± 9.83	**3.61 (1.50–5.73)**	**0.001**	**2.91 (0.89–4.93)**	**0.005**
3 times per week or more	153	7.69 ± 10.10	**4.05 (1.73–6.38)**	**0.001**	**2.84 (0.61–5.07)**	**0.013**
**Amount of total egg consumption, 1 egg/week increment**			0.13 (−0.05–0.32)	0.162	0.12 (−0.06–0.30)	0.193
**Frequency of whole egg consumption**						
Never	136	4.26 ± 7.93	Reference		Reference	
1–3 times per month	310	5.94 ± 9.08	1.69 (−0.19–3.56)	0.078	1.65 (−0.14–3.44)	0.070
1 time per week	119	7.88 ± 9.99	**3.63 (1.34–5.91)**	**0.002**	**3.21 (1.05–5.38)**	**0.004**
2 times per week or more	256	7.13 ± 9.93	**2.87 (0.94–4.81)**	**0.004**	**2.66 (0.82–4.51)**	**0.005**
**Amount of whole egg consumption, 1 egg/week increment**			0.10 (−0.09–0.30)	0.288	0.12 (−0.06–0.31)	0.194
**Frequency of egg white consumption**						
Never	755	6.32 ± 9.38	Reference		Reference	
1–3 times per month	39	5.97 ± 8.74	−0.35 (−3.36–2.67)	0.822	−2.26 (−5.16–0.64)	0.126
1 time per week or more	26	6.18 ± 10.15	−0.14 (−3.80–3.52)	0.940	−1.15 (−4.69–2.39)	0.524
**Amount of egg white consumption, 1 egg/week increment**			0.01 (−0.70–0.72)	0.983	−0.24 (−0.95–0.46)	0.500
**Frequency of egg substitute consumption**						
Never	774	6.27 ± 9.28	Reference		Reference	
Ever consumption	47	7.09 ± 10.81	0.83 (−1.93–3.58)	0.557	−0.93 (−3.64–1.77)	0.499
**Amount of egg substitute consumption, 1 egg/week increment**			**1.81 (0.49–3.14)**	**0.007**	0.72 (−0.60–2.03)	0.285
**Frequency of eggs with fat consumption**						
Never	311	5.95 ± 9.09	Reference		Reference	
1–3 times per month	272	6.49 ± 9.49	0.54 (−0.99–2.06)	0.490	1.31 (−0.17–2.79)	0.082
1 time per week	78	5.34 ± 8.52	−0.61 (−2.93–1.71)	0.605	−0.11 (−2.32–2.10)	0.922
2 times per week or more	160	7.21 ± 10.07	1.26 (−0.52–3.04)	0.166	**2.06 (0.31–3.81)**	**0.021**
**Amount of eggs with fat consumption, 1 egg/week increment**			−0.05 (−0.27–0.17)	0.635	0.04 (−0.17–0.26)	0.708
**Frequency of egg salad consumption**						
Never	657	6.27 ± 9.42	Reference		Reference	
1–3 times per month	136	6.24 ± 9.13	−0.02 (−1.75–1.71)	0.979	0.20 (−1.46–1.86)	0.813
1 time per week or more	26	7.88 ± 9.75	1.61 (−2.06–5.28)	0.390	2.76 (−0.77–6.30)	0.125
**Amount of egg salad consumption, 1 egg/week increment**			0.38 (−0.55–1.30)	0.426	0.61 (−0.28–1.49)	0.180

SD, standard deviation; CI, confidence interval. * Significant *p*-values (<0.05) are in bold. ** Adjusted for household poverty level, maternal pre-pregnancy body mass index, Healthy Eating Index, race, education, employment status, enrollment in the Special Supplemental Nutrition Program for Women, Infants, and Children, region of residency, and cigarette smoking status during pregnancy.

## Data Availability

The data described in the manuscript, code book, and analytic code will not be made available because the data were provided by the CDC and cannot be shared without permission. Researchers who are interested in using the IFPS II data can contact the CDC directly (ifps@cdc.gov).

## Abbreviations

The following abbreviations are used in this manuscript:

SIDS	sudden infant death syndrome
AAP	American Academy of Pediatrics
ACOG	American College of Obstetricians and Gynecologists
IFPS II	Infant Feeding Practices Study II
FDA	U.S. Food and Drug Administration
CDC	Centers for Disease Control and Prevention
Y6FU	Year 6 Follow-Up
WIC	Special Supplemental Nutrition Program for Women, Infants, and Children
BMI	body mass index
DHQ	Diet History Questionnaire
HEI	Healthy Eating Index
USDA	U.S. Department of Agriculture
SD	standard deviation
ANOVA	analysis of variance
LSD	least significant difference
OR	odds ratio
CI	confidence interval
RDA	Recommended Dietary Allowance
LCPUFA	long-chain polyunsaturated fatty acids

## Appendix A

**Table A1:** Comparison of socio-demographic and pregnancy-related characteristics between the analytic and excluded samples.

	Analytic Sample (N = 1039)		Excluded Sample (N = 3863)		
Characteristics *	n (%)	Mean ± SD	n (%)	Mean ± SD	*p*-Value **
**Maternal age, years**		29.02 ± 5.34		27.97 ± 5.80	**<0.001**
**% of Federal poverty level**		262.10 ± 189.30		243.90 ± 199.00	**0.007**
**Pre-pregnancy BMI, kg/m^2^**		26.28 ± 6.45		26.47 ± 6.97	0.396
**Maternal race/ethnicity**					
Non-Hispanic White	880 (85.2)		2983 (80.2)		**0.001**
Non-Hispanic Black	40 (3.9)		260 (7.0)		
Hispanic	66 (6.4)		269 (7.2)		
Non-Hispanic Asian/Pacific Islander/Other	47 (4.6)		209 (5.6)		
**Maternal highest education level**					**<0.001**
High school or lower	187 (18.9)		869 (26.4)		
1–3 years of college	388 (39.2)		1369 (41.6)		
College graduate	309 (31.2)		781 (23.8)		
Postgraduate	105 (10.6)		270 (8.2)		
**Maternal employment status**					
Unemployed	364 (35.2)		1271 (33.1)		0.207
Employed	671 (64.8)		2571 (66.9)		
**Household size**					
1–2 people	260 (25.0)		1051 (27.2)		**0.008**
3 people	400 (38.5)		1275 (33.0)		
4 people	217 (20.9)		839 (21.7)		
≥5 people	162 (15.6)		698 (18.1)		
**Annual household income level**					
<$25,000	220 (21.2)		985 (25.5)		**0.001**
$25,000 ≤ $39,999	228 (21.9)		929 (24.1)		
$40,000 ≤ $59,999	243 (23.4)		858 (22.2)		
≥$60,000	348 (33.5)		1091 (28.2)		
**Maternal WIC recipient status**					
Non-recipient	619 (59.6)		2042 (52.9)		**<0.001**
Recipient	420 (40.4)		1821 (47.1)		
**Region of residency**					
New England	52 (5.0)		154 (4.0)		0.052
Middle Atlantic	122 (11.7)		497 (12.9)		
East North Central	225 (21.7)		758 (19.6)		
West North Central	96 (9.2)		332 (8.6)		
South Atlantic	153 (14.7)		698 (18.1)		
East South Central	56 (5.4)		245 (6.3)		
West South Central	117 (11.3)		435 (11.3)		
Mountain	107 (10.3)		319 (8.3)		
Pacific	111 (10.7)		425 (11.0)		
**Maternal cigarette smoking during pregnancy**					
No	940 (91.0)		3341 (87.1)		**0.001**
Yes	93 (9.0)		496 (12.9)		

BMI, body mass index; WIC, Special Supplemental Nutrition Program for Women, Infants, and Children; SD, standard deviation. * For some characteristics, the sum of categories is less than the total due to missing data. ** Significant *p*-values (<0.05) are in bold.

**Table A2:** Variables of potential confounders.

Potential Confounder	Type of Variable	Unit or Categories
Age	Continuous	Years
Healthy Eating Index	Continuous	Scoring unit
Pre-pregnancy body mass index	Continuous	Kg/m^2^
Race/ethnicity	Categorical	Non-Hispanic White, non-Hispanic Black, Hispanic, and Non-Hispanic Asian/Pacific Islander/other
The highest education level	Categorical	High school or lower, 1–3 years college, college graduate, and postgraduate
Employment status	Categorical	Unemployed and employed
Household size	Categorical	1–2, 3, 4, and ≥5 people
Annual household income	Categorical	<$25,000, $25,000 to $39,999, $40,000 to $59,999, and ≥$60,000
WIC recipient status	Categorical	Non-recipient and recipient
Region of residency	Categorical	New England, Middle Atlantic, East North Central, West North Central, South Atlantic, East South Central, West South Central, Mountain, and Pacific
Smoking status during pregnancy	Categorical	Yes and no

**Table A3:** Breastfeeding duration by socio-demographic and pregnancy-related characteristics.

	Any Breastfeeding Duration, Weeks		Exclusive Breastfeeding Duration, Weeks	
Characteristics *	Mean ± SD	*p*-Value **	Mean ± SD	*p*-Value **
**Overall**	26.43 ± 20.69		6.31 ± 9.37	
**Maternal age, years (mean difference ± SE) *****	1.09 ± 0.09	**<0.001**	0.14 ± 0.03	**<0.001**
**100% of Federal poverty level (mean difference ± SE) *****	1.10 ± 0.26	**<0.001**	0.20 ± 0.08	**0.017**
**Pre-pregnancy BMI, kg/m^2^ *****	−0.28 ± 0.08	**<0.001**	−0.13 ± 0.02	**<0.001**
**Maternal race/ethnicity**		**<0.001**		**<0.001**
Non-Hispanic White	25.42 ± 26.49		5.79 ± 8.91	
Non-Hispanic Black	16.82 ± 21.87		1.59 ± 4.27	
Hispanic	20.91 ± 23.83		3.53 ± 6.63	
Non-Hispanic Asian/Pacific Islander/Other	27.11 ± 24.45		4.37 ± 8.18	
**Maternal highest education level**		**<0.001**		**<0.001**
High school or lower	15.76 ± 20.22		3.32 ± 6.51	
1–3 years of college	23.17 ± 24.86		5.02 ± 8.29	
College graduate	31.97 ± 26.35		7.06 ± 9.59	
Postgraduate	36.78 ± 30.81		8.09 ± 10.48	
**Maternal employment status**		**<0.001**		**<0.001**
Unemployed	27.17 ± 28.26		6.24 ± 9.44	
Employed	23.30 ± 24.70		4.87 ± 8.11	
**Household size**		0.599		0.723
1–2 people	24.30 ± 27.69		5.07 ± 8.44	
3 people	25.39 ± 24.42		5.54 ± 8.62	
4 people	23.58 ± 25.52		5.28 ± 8.59	
≥5 people	25.02 ± 28.06		5.26 ± 8.80	
**Annual household income level**		**<0.001**		**<0.001**
<$25,000	17.65 ± 22.96		4.01 ± 7.63	
$25,000 ≤ $39,999	23.56 ± 25.42		5.33 ± 8.51	
$40,000 ≤ $59,999	28.14 ± 27.71		6.09 ± 9.21	
≥$60,000	27.49 ± 26.53		5.59 ± 8.69	
**Maternal WIC recipient status**		**<0.001**		**0.008**
Non-recipient	27.23 ± 26.70		5.68 ± 8.85	
Recipient	21.03 ± 24.90		4.80 ± 8.19	
**Region of residency**		**<0.001**		**<0.001**
New England	24.64 ± 26.80		5.07 ± 8.27	
Middle Atlantic	23.85 ± 25.97		4.19 ± 7.97	
East North Central	23.66 ± 27.91		5.01 ± 8.38	
West North Central	24.28 ± 22.60		5.88 ± 8.72	
South Atlantic	22.48 ± 26.15		4.59 ± 7.97	
East South Central	18.67 ± 22.84		4.63 ± 8.30	
West South Central	22.44 ± 25.17		4.81 ± 8.26	
Mountain	29.29 ± 20.96		6.72 ± 9.20	
Pacific	32.50 ± 29.82		7.56 ± 9.92	
**Maternal cigarette smoking during pregnancy**		**<0.001**		**<0.001**
No	26.32 ± 26.37		5.71 ± 8.85	
Yes	10.29 ± 18.68		1.92 ± 4.62	
**Child sex**		0.402		0.128
Male	25.10 ± 26.97		5.07 ± 8.41	
Female	24.24 ± 25.32		5.56 ± 8.76	

BMI, body mass index; WIC, Special Supplemental Nutrition Program for Women, Infants, and Children; SD, standard deviation; SE, standard error. * For some characteristics, the sum of categories is less than the total due to missing data. ** *p*-values from analysis of variance for categorical correlates and linear regression models for continuous correlates. Significant *p*-values (<0.05) are in bold. *** Mean difference in breastfeeding duration per 1-unit increment in the correlate.
